# Oncolytic adenovirus improves anti-tumor efficacy of adoptive T cell therapy by breaking tumor-induced immunosuppression and peripheral tolerance

**DOI:** 10.1186/2051-1426-3-S2-P315

**Published:** 2015-11-04

**Authors:** Siri Tähtinen, Susanna Grönberg-Vähä-Koskela, Dave Lumen, Maiju Merisalo-Soikkeli, Mikko Siurala, Anu J Airaksinen, Markus Vähä-Koskela, Akseli Hemminki

**Affiliations:** 1 Cancer Gene Therapy Group, Medicum, Haartman Institute, University of Helsinki, Helsinki, Finland; 2 Laboratory of Radiochemistry, Department of Chemistry, University of Helsinki, Helsinki, Finland; 3 Cancer Gene Therapy Group, Medicum, Haartman Institute, University of Helsinki, Helsinki, Finland and TILT Biotherapeutics Ltd, Helsinki, Finland

## 

Adoptive T cell therapies (ACT) using genetically re-directed blood T cells have shown exceptional efficacy in the treatment of CD19-expressing hematological malignancies. In contrast, rapid progress in the development of approaches to treat solid tumors have provided proof-of-concept data but modest response rates due to several immune evasion mechanisms, which contribute to tumor tolerance. Oncolytic adenoviruses are conditionally replicating, cancer cell lysing viruses that are intrinsically immunogenic due to inherent pathogen-associated molecular patterns. We hypothesized that we could employ the tremendous immunostimulatory capacity of oncolytic adenoviruses to break tumor-associated tolerance to T cells and set out to test this in a highly resistant B16.OVA murine melanoma model.

Following adoptive transfer of T cell receptor (TCR) engineered SIINFEKL-specific, CD8a+ enriched OT-I lymphocytes, control of B16.OVA tumor growth was superior in adenovirus (Ad) treated immunocompetent C57BL/6 mice compared to control mice, even in the absence of active oncolysis. Significant increase in tumor-infiltrating immune cells such as CD45^+^ leukocytes, CD8^+^ lymphocytes and F4/80^+^ macrophages was seen Ad-treated tumors, suggesting enhanced tumor immunogenicity. Surprisingly, no statistically significant difference in numbers of tumor-infiltrating OT-I cells was detected between treatment groups either by *in vivo* SPECT/CT imaging or by *ex vivo* flow cytometry. Instead, adenovirus infection resulted in a pro-inflammatory tumor microenvironment via upregulation of cytokines and chemokines including IFN-γ, CCL2, CCL3 and CCL5. In addition, Intratumoral administration of adenovirus induced maturation of professional CD11c^+^ antigen-presenting cells (APCs) both in tumors and in tumor-draining lymph nodes. Interactions between APCs and T cells were further supported by increased levels of CD45-CD31-gp38+ fibroblastic reticular cells (FRCs) in dissociated tissues of Ad-treated mice compared to control groups. Subsequently, increased numbers of activated, IFN-γ+ tumor-infiltrating CD8+ T cells (TILs) were detected in Ad-treated mice, suggesting that tumor-induced T cell hypofunction was overcome. Finally, an increase in endogenous CD8+ TILs specific for tumor-associated antigens TRP-2 and gp100 was detected in combination treated mice, indicating repertoire expansion following immunotherapy. Majority of these adenovirus/ACT -treated mice rejected the re-challenge of parental B16.F10 tumors, suggesting that systemic, endogenous anti-tumor immunity was induced despite local (intratumoral) injection of adenovirus.

In conclusion, combining ACT with oncolytic adenovirus can break both local immunosuppression and peripheral tolerance of tumor-specific T cells, leading to systemic anti-tumor immunity and enhanced therapeutic efficacy. Importantly, these two modalities are not merely an attractive combination, but could represent a way to achieve “CD19-like” results in the treatment of solid tumors.

